# Novel Methodology for Rapid Detection of *KRAS* Mutation Using PNA-LNA Mediated Loop-Mediated Isothermal Amplification

**DOI:** 10.1371/journal.pone.0151654

**Published:** 2016-03-21

**Authors:** Masahiro Itonaga, Ibu Matsuzaki, Kenji Warigaya, Takaaki Tamura, Yuki Shimizu, Masakazu Fujimoto, Fumiyoshi Kojima, Masao Ichinose, Shin-ichi Murata

**Affiliations:** 1 Department of Human Pathology, Wakayama Medical University, Wakayama, Japan; 2 Second Department of Internal Medicine, Wakayama Medical University, Wakayama, Japan; New York University, UNITED STATES

## Abstract

Detecting point mutation of human cancer cells quickly and accurately is gaining in importance for pathological diagnosis and choice of therapeutic approach. In the present study, we present novel methodology, peptide nucleic acid—locked nucleic acid mediated loop-mediated isothermal amplification (PNA-LNA mediated LAMP), for rapid detection of *KRAS* mutation using advantages of both artificial DNA and LAMP. PNA-LNA mediated LAMP reactions occurred under isothermal temperature conditions of with 4 primary primers set for the target regions on the *KRAS* gene, clamping PNA probe that was complimentary to the wild type sequence and LNA primers complementary to the mutated sequences. PNA-LNA mediated LAMP was applied for cDNA from 4 kinds of pancreatic carcinoma cell lines with or without *KRAS* point mutation. The amplified DNA products were verified by naked-eye as well as a real-time PCR equipment. By PNA-LNA mediated LAMP, amplification of wild type *KRAS* DNA was blocked by clamping PNA probe, whereas, mutant type *KRAS* DNA was significantly amplified within 50 min. Mutant alleles could be detected in samples which diluted until 0.1% of mutant-to-wild type ratio. On the other hand, mutant alleles could be reproducibly with a mutant-to-wild type ratio of 30% by direct sequencing and of 1% by PNA-clamping PCR. The limit of detection (LOD) of PNA-LNA mediated LAMP was much lower than the other conventional methods. Competition of LNA clamping primers complementary to two different subtypes (G12D and G12V) of mutant *KRAS* gene indicated different amplification time depend on subtypes of mutant cDNA. PNA-LNA mediated LAMP is a simple, rapid, specific and sensitive methodology for the detection of *KRAS* mutation.

## Introduction

Somatic point mutations of human cancers have been studied in basic and clinical research of tumorigenesis, for pathological diagnosis and for clinical therapies [1, 2]. For example, *KRAS* point mutations in codon 12 or 13 occur in 35–50% of colorectal cancers and in 80–90% of pancreatic cancers [3, 4, 5]. *KRAS* point mutations have been studied in terms of pathogenesis and progression in colorectal cancers and their identification has recently been shown to be clinically useful as an indicator of cetuximab therapeutic effect, represented by overall survival and progression-free survival [6]. The combination of cytological analysis with *KRAS* mutation status improves diagnostic accuracy for cytological diagnosis of pancreatic cancers by endoscopic ultrasound-guided fine-needle aspiration biopsy (EUS-FNAB) [7].

There are various methods available for the detection of point mutations of cancer cells including PCR direct sequencing, multiplex PCR and array hybridization, and amplification refractory mutation systems. A direct sequencing method is the most popular method to molecularly characterize genetic variants because it provides the means to detect all potential variations, including base substitutions, insertions and deletions. However, this method has some limitations when applied to clinical samples. One important limitation is that direct sequencing is not sensitive enough (10–30% level) to detect a specific point mutation [8]. Since clinical samples such as EUS-FNAB may include a mixture of cancer cells with mutated and non-neoplastic cells, the method used to analyze point mutations in such clinical samples needs to be of high sensitivity. Moreover, application of a direct sequencing method to cytological diagnosis in hospitals involves complicated procedures, long analytical times (about 6 hours) and expensive equipment. Therefore, for the detection of point mutations, there is a need to develop a simple, rapid, specific and sensitive methodology.

In basic research, much attention has recently been paid to artificial DNAs such as PNA and LNA because of their characteristic of high sequence specificity. PNA is a synthetic DNA analog in which the normal phosphodiester backbone is replaced with a 2-aminoethylglycine chain. The nucleobases of PNA complement DNA or RNA with the normal A-T and G-C geometry [9, 10, 11]. LNA, which is another DNA/RNA analog, has a special ribonucleoside structure in which the 2’-oxygen and the 4’-carbon atoms are connected with a methylene bridge. A PNA-clamping PCR method has been developed for mutation detection based on the following principles [12]. The melting temperature (Tm) of a perfectly matched PNA-DNA duplex is much higher than that of a perfectly matched DNA-DNA duplex of the same length. A single mismatched PNA-DNA duplex has a Tm that is 10–18°C lower than that of a perfectly matched PNA-DNA duplex [13]. This large Tm difference between perfectly matched and mismatched PNA-DNA hybrids makes PNA a good sensor of point mutations. In addition, PNA cannot serve as a primer for polymerization, nor can it function as a substrate for exonuclease activities of Taq polymerase. Therefore, PNA on a perfectly matched template DNA can specifically block the reactions of primer annealing or chain elongation in PCR [14, 15, 16]. PNA-clamping PCR provides enhanced sensitivities compared with direct sequencing [8]. Using a combination of a clamping PNA probe and an LNA primer, Nagai Y et al. successfully detected genetic heterogeneity of epidermal growth factor receptor (*EGFR*) in non-small cell lung cancer [17].

LAMP was developed as a non-PCR DNA amplification method [18]. The LAMP reactions occur under an isothermal temperature condition of around 60 to 65°C in a reaction solution that includes a Bst DNA polymerase with strand-displacement activity and a set of 4 or 6 primers designed to amplify 6 or 8 distinct regions of the target DNA. The reaction is completed within 15 to 50 min. The LAMP products can be quantitatively assessed by measuring the fluorescence intensity from DNA-intercalating fluorophores such as SYBR Green I and YO-PRO-1 [19] using real-time PCR equipment or by measuring the turbidity of the magnesium pyrophosphate produced during the LAMP reactions using a turbidimeter. One advantage of this method is that the amplified DNA product can be visualized by adding metal ion indicators such as calcein [20] and hydroxynaphthol blue (HNB) [21]. LAMP assay has recently been employed to identify microorganisms such as bacteria [22] and viruses [23, 24], and to detect lymph node metastasis of cancer cells [25, 26]. However, there have been few reports of application of the LAMP method to detection of DNA point mutations [27, 28, 29].

In this report, in order to detect *KRAS* point mutations in pancreatic cancer cells we developed a novel, non-PCR molecular method, PNA-LNA mediated LAMP, which takes advantage of both LAMP technology and high sequence specific artificial nucleic acids.

## Materials and Methods

### Cell lines

Four pancreatic carcinoma cell lines were used: BxPC-3-Luc#2 (Bx) and Hs766T, which express the wild type *KRAS* gene, and Panc-1 and QGP-1, which express mutant type *KRAS* genes. Panc-1 expresses *KRAS* c.35G>A (p.G12D) and QGP-1 expresses *KRAS* c.35G>T (p.G12V) mutation of codon 12 of the *KRAS* gene (NC_000012). Bx and QGP-1 were purchased from the JCRB cell bank. Hs766T and Panc-1 were purchased from the ATCC and RIKEN BRC respectively, through the National Bio-Resource Project of the MEXT. Cells were grown in 10 cm culture dishes. Bx, QGP-1 and Panc-1 cells were maintained in RPMI1640 with 10% heat inactivated fetal bovine serum. Hs766T was maintained in DMEM with 10% heat inactivated fetal bovine serum.

### Preparation of genomic cDNA

RNA was isolated from cell lines using the RNeasy Mini Kit (QIAGEN, Hilden, Germany) and was converted to cDNA using the GoScript Reverse Transcription System (Promega, USA).

### PCR direct sequencing analysis

Mutational analysis of *KRAS* was performed using cDNA that evaluated exon 2 of *KRAS* using the following primers: *KRAS* forward primer: 5’- ATTTCGGACTGGGAGCGAGC-3’, and *KRAS* reverse primer: 5’- GTCCCTCATTGCACTGTACTC-3’. PCR amplification was performed using a mixture that contained 400 nM of each primer, 200 nM dNTPs and 0.0325 u/μl Go Taq DNA polymerase (Promega, USA). PCR cycling conditions were 95°C for 5 min followed by 35 cycles of 95°C for 30 sec, 60°C for 30 sec and 72°C for 30 sec, and then 72°C 4 minutes. The PCR products were subjected to 2% agarose gel electrophoresis at 100 V, and the gel was checked for amplified fragments. PCR fragments were cleaned with QIAquick Spin (Qiagen, Hilden, Germany). Sequencing was performed using the BigDye Terminator version 3.1 Cycle sequencing kit (Life Technologies, USA) and the 3500 Genetic analyzer (Life Technologies, USA), and sequences were analyzed in both sense and antisense directions.

### PNA-clamping real-time PCR

PCR amplification was performed in a total volume of 20 μl, containing 0.08 ng cDNA, 1×Fast Real-Time PCR SYBR Green PCR master mix (Bio-Rad Laboratories, USA), 400 nM of each primer and 200 nM PNA (Panagene, Korea). PCR cycling conditions were 95°C for 5 min followed by three-step cycling conditions: 40 cycles of 95°C for 10 sec, 70°C for 10 sec and 60°C for 20 sec, followed by a melting curve from 60 to 95°C. PCR was performed using the forward primer 5’- GCCTGCTGAAAATGACTGAATATAA -3’, the reverse primer 5’-CGTCAAGGCACTCTTGCCTAC-3’, and the clamping PNA probe TACGCCACCAGCTCC.

### The PNA-LNA mediated LAMP method

#### 1) Principle of PNA-LNA mediated LAMP

**Fig 1:**
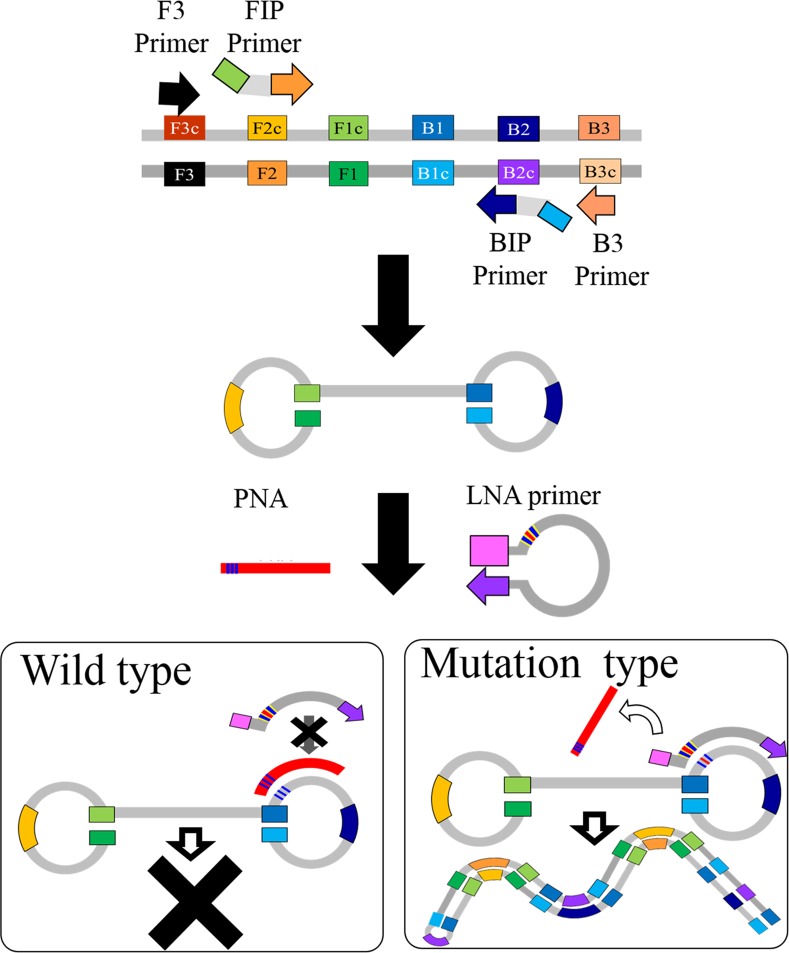
Principle of PNA-LNA mediated LAMP. When the target gene is wild-type, the clamping PNA probe forms a stable duplex with the dumbbell structure, and interferes with the annealing and extension of the LNA primer. On the other hand, when the target gene is mutated, the clamping PNA probe does not anneal with the cDNA because of the single-base mismatch, and the LNA primer breaks its internal interaction to bind the target, and the extension reaction proceeds.

The principle of PNA-LNA mediated LAMP is shown in Fig 1. The LAMP reactions are based on a set of 4 primary primers that were specifically designed to recognize 6 distinct regions on the target gene: a pair of outer primers (F3 and B3) and a pair of inner primers (FIP and BIP) that have a tag complementary to a downstream region in the opposite strand of the target (F1 and B1). In PNA-LNA mediated LAMP, a clamping PNA probe specific for the wild type nucleotide and additional LNA primers complementary to the mutant type nucleotide are designed for the looped region of the primary LAMP products. The reactions are conducted at **a** constant temperature of 65°C in the presence of a strand displacement DNA polymerase. The FIP primer anneals and extends on the target DNA and the newly synthesized DNA chains are displaced by extension of F3. Similarly, the BIP primer anneals and extends on target DNA and the newly synthesized DNA chains are displaced by extension of B3. The displaced products generate stem loop structures which represent the starting structure for the LAMP reactions. If the target gene is wild-type, the clamping PNA probe forms a stable duplex with the dumbbell structure and interferes with the annealing and extension of the LNA primer. If the target gene is mutated, the PNA does not clamp since there is a single-base mismatch. Instead of PNA, the LNA primer binds and anneals to the target region and consequently starts extending.

#### 2) Primer design for PNA-LNA mediated LAMP (Fig 2)

A set of four primary LAMP primers (F3, FIP, B3 and BIP) were designed using PrimerExplorer V4 (Eiken Chemical, Japan) and manually added optimization for LAMP reactions quickly. A clamping PNA probe was designed that was complementary to a sequence including the wild type nucleotide and LNA primers were designed that were complementary to a sequence including the mutated nucleotides (LNAG12D and LNAG12V). PNA- and LNA-containing oligo DNAs were obtained from PANAGENE (Korea) and EXIQON (Denmark), respectively.

**Fig 2:**
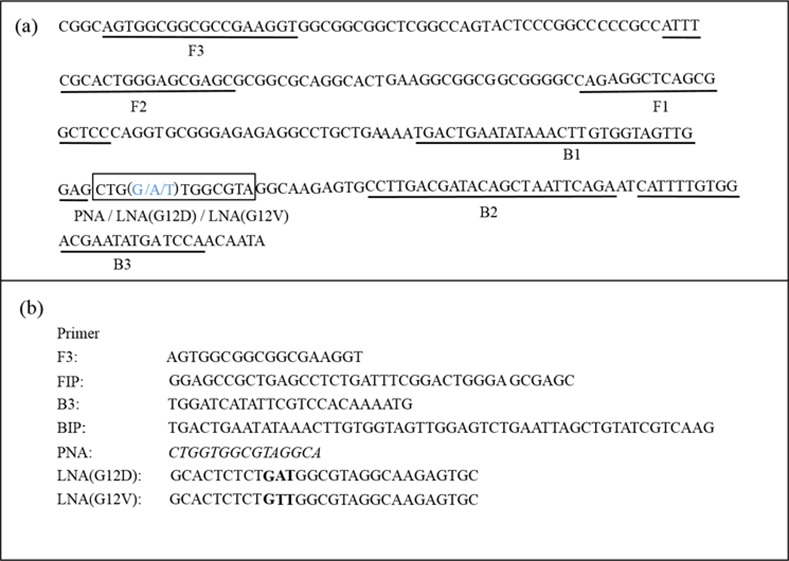
Set of PNA-LNA mediated LAMP primers used. (a) The figure shows the DNA sequence of codon 12 of the *KRAS* gene. The wild and mutated nucleotides in *KRAS* codon 12 are shown in bold blue type (G for the wild type, A for G12D and T for G12V.). (b) The figure indicates the design of the set of LAMP primers and the PNA probe. The italic faces indicate modification site of PNA and the bold faces indicate modification site of LNA.

#### 3) Reaction of PNA-LNA mediated LAMP

The PNA-LNA mediated LAMP method was applied to genomic cDNA from 2 wild type KRAS cell lines (Bx and HS766T) and 2 mutant type *KRAS* cell lines (Panc-1 and QGP-1). PNA-LNA mediated LAMP was performed in a mixture containing 8 units of Bst DNA polymerase (Nippon Gene, Japan), a 1.4 mM dNTPs mixture (Nippon Gene, Japan), 6.25 nM YO-PRO-1 (Life Technology, USA), and the primers (1.6 μM FIP, 1.6 μM BIP, 0.2 μM F3, 0.2 μM B3, 0.8 μM LNA, 0.25 μM PNA). Both a positive control sample without a clamping PNA probe and a negative control sample without cDNA were included in each run. The LNA primer complementary to the G12D mutant gene was used for Bx and Panc-1 cDNA, and the LNA primer complementary to the G12V mutant gene was used for HS766T and QGP-1 cDNA. Components for the reaction were maintained at 4°C until amplification was initiated by heating to 65°C for 50 min and then terminated by heating at 95°C for 5 min.

#### 4) Detection and evaluation of PNA-LNA mediated LAMP products

LAMP products were detected using real-time PCR equipment (MyIQ, BioRad, USA), by agarose gel electrophoresis or by the naked-eye. Since the LAMP reactions occurred under isothermal conditions, real-time PCR equipment was used as a fluorometer for quantitative evaluation. The variation of relative fluorescence units (RFU) was analyzed using the threshold time (TT) value for each tested sample. The TT value was defined as the time it took for fluorescence intensity to reach a threshold value of 100 after baseline subtraction. ΔTT was defined as the TT value obtained using the clamping PNA probe minus the TT value obtained without the clamping PNA probe. LAMP products were detected by 1.0% agarose gel in 1×TAE. LAMP products were also visualized and detected or by adding calcein (0.127 μg/ml) before the start of the reactions. After stopping the reactions, the fluorescence from the products in the tubes were observed under irradiation by UV light. Green fluorescence similar to the positive control was assessed as a positive result, and no fluorescence similar to the negative control was assessed as a negative result.

### Evaluation of the limit of detection (LOD) of PNA-LNA mediated LAMP

To evaluate the LOD of the PNA-LNA mediated LAMP method with a low concentration of the *KRAS* mutated gene, a dilution assay was performed using cDNA samples from the Panc-1 mutant cell line and the QGP-1 mutant cell line that were serially diluted with wild-type cDNA from the Bx cell line and HS766T cell line (100, 50, 10, 1, 0.5, 0.1 and 0%).

### Detection of *KRAS* mutation subtypes with PNA-LNA mediated LAMP

To detect mutation subtypes in codon 12 of the *KRAS* gene, two different LNA primers, LNA (G12D) and LNA (G12V), were used in the PNA-LNA mediated LAMP. The sequence of the LNA (G12D) and LNA (G12V) primers was complementary to that of sequences containing mutations in codon 12 of the mutant *KRAS* gene. The PNA-LNA mediated LAMP assay was performed 10 times using the same cDNA samples from the cell lines, Panc-1 and QGP-1, which express the G12D and G12V *KRAS* mutations, respectively.

For quantitative evaluation, ΔTT was used.

### Reproducibility of PNA-LNA mediated LAMP

More than three times were tested to confirm the reproducibility of PNA-LNA mediated LAMP using independent cDNA samples.

### Statistical analysis

Categorical data analysis was conducted using the t test with SPSS version 11.0 (SPSS).

All differences were considered statistically significant if the *p* value was < .05.

## Results

### Detection of *KRAS* point mutation by PNA-LNA mediated LAMP

**Fig 3:**
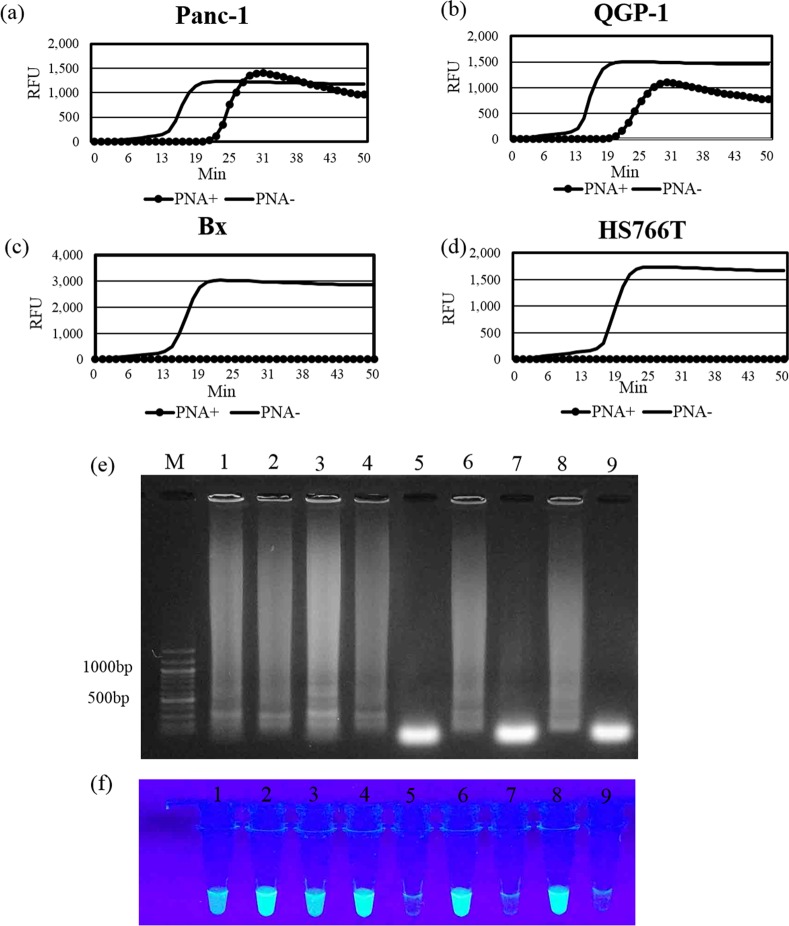
Amplification products of *KRAS* by PNA-LNA mediated LAMP. (a-d) The graphs indicate the fluorescence intensity of the LAMP products as measured using real-time PCR equipment. (e) The figure shows the detection by agarose gel electrophoresis. (f) The fluorescence emitted from LAMP products in microtubes under UV light was visually assessed. LNA primer complementary to the G12D mutant gene was used for Panc-1 and Bx cDNA samples, and the LNA primer complementary to the G12V mutant gene was used for QGP-1 and HS766T cDNA samples. M: size maker, lane 1–8: LAMP products from Panc-1 with PNA, Panc-1 without PNA, QGP-1 with PNA, QGP-1 without PNA, Bx with PNA, Bx without PNA, HS766T with PNA and HS766T without PNA, lane 9: negative control without cDNA.

In the LAMP assay without a PNA clamping probe, LAMP products were detected in all of the samples analyzed from the cell lines Bx, HS766T, Panc-1 and QGP-1, using both real-time PCR equipment over 50 min and analysis by the agarose gel electrophoresis and the naked eye at 50 min (Fig 3).

In the LAMP assay with a clamping PNA probe, LAMP products were produced in both of the samples obtained from the mutant cell lines (Panc-1 and QGP-1) (Fig 3A and 3B), even though amplification time was delayed compared with that of PNA negative samples. In contrast, in the LAMP assay with a clamping PNA probe, amplification of cDNA was completely blocked in all samples from the wild type cell lines, Bx and HS766T (Fig 3C and 3D).

The LAMP products were separated on agarose gel electrophoresis to determine specificity. The ladder-like DNA bands were detected in all samples in the LAMP assay without a clamping PNA probe. However, in the LAMP assay with a PNA clamping probe, the ladder-like DNA bands were only detected in the samples from Panc-1 and QGP-1 cells, whereas the samples from the Bx and HS766T cell lines showed no bands (Fig 3E).

### Visual detection of *KRAS* point mutation by PNA-LNA mediated LAMP

In the samples to which a fluorescent detection reagent had been added, LAMP products were observed with the naked eye under UV light after 50 min reaction time. The results obtained were similar to those obtained by detection using PCR equipment. Thus, bright fluorescence was detected in all samples in the LAMP assay without a clamping PNA probe. However, in the LAMP assay with a PNA clamping probe, bright fluorescence was only detected in the samples from Panc-1 and QGP-1 cells, whereas the samples from the Bx and HS766T cell lines showed no fluorescence (Fig 3F).

### The LOD of *KRAS* point mutation by PNA-LNA mediated LAMP, direct sequencing and PNA-clamping PCR

**Fig 4:**
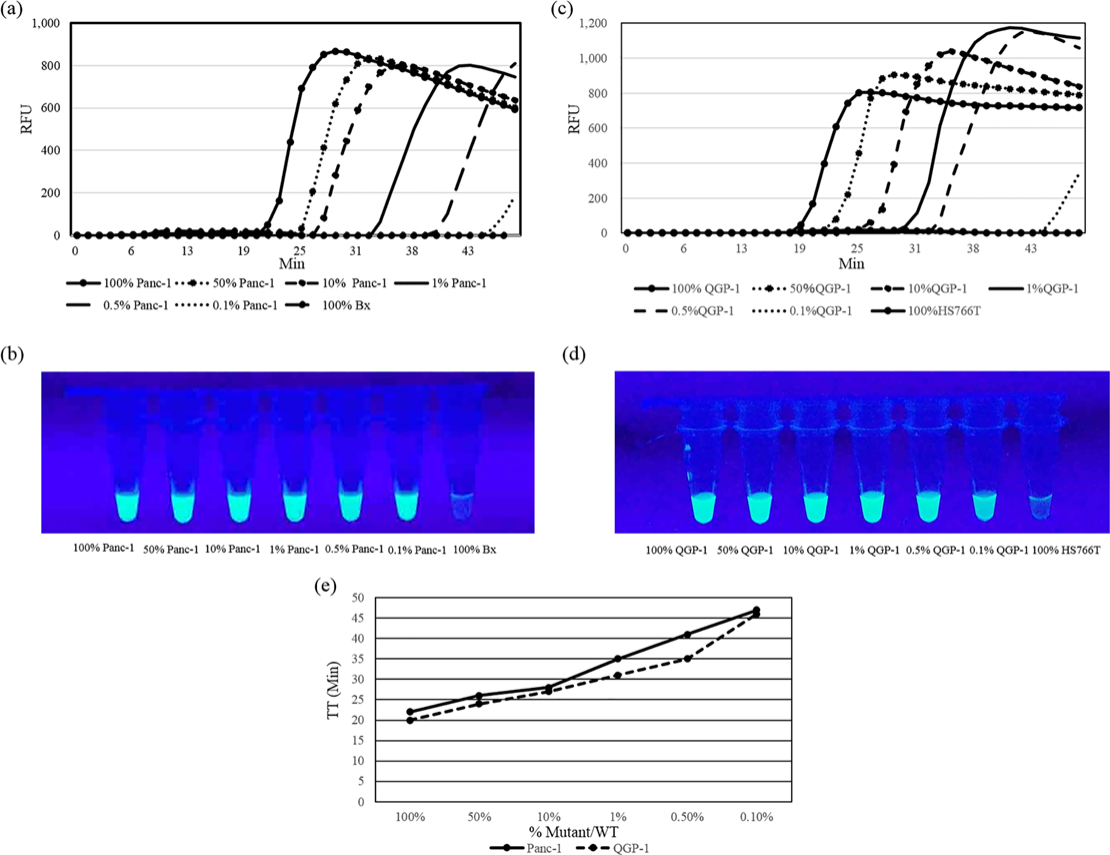
The LOD of PNA-LNA mediated LAMP. Mutant type Panc-1 and QGP-1 samples were serially diluted with wild type Bx and HS766T samples (100% Bx and 100% HS766T) to give Panc-1 and QGP-1 cDNA samples concentrations of 100, 10, 1, 0.5, 0.1 and 0%, which were then analyzed using PNA-LNA mediated LAMP. As a negative control, we use 100% Bx and 100% HS766T cDNA samples. (a, c) The graph shows the fluorescence intensity of LAMP products measured using real-time PCR equipment. (b, d) The fluorescence emitted by LAMP products in microtubes under UV light was visually assessed. (e) The graph shows the respective graph of TT vs %Mutant/ WT for both mutations. The TT value was defined as the time it took for fluorescence intensity to reach a threshold value of 100 after baseline subtraction.

For the LOD, the sensitivity of *KRAS* point mutation detection by PNA-LNA mediated LAMP was determined using a dilution assay in which mutant KRAS cDNA from Panc-1 cells and QGP-1 cells was diluted to 50, 10, 1, 0.5, and 0.1% with wild type cDNA from Bx cells and HS766T cells. The LAMP products were detected using both real-time PCR equipment and the naked eye. In this dilution assay, PNA-LNA mediated LAMP amplified mutant *KRAS* cDNA within 50 min in all Panc1 samples but not in the wild type control Bx cells (Fig 4A and 4B). Similarly, PNA-LNA mediated LAMP amplified mutant *KRAS* cDNA within 50 min in all QGP-1 samples but not in the wild type control HS766T cells (Fig 4C and 4D). The graph shows the respective graph of TT vs %Mutant/ WT for both mutations (Fig 4E).Mutant alleles could be reproducibly detected within 50 min in samples diluted down to a mutant-to-wild type ratio of 0.1% for both mutations.

Additionally, the LOD of *KRAS* point mutation by PNA-LNA mediated LAMP was compared with other conventional methods including a direct sequencing assay and PNA clamping PCR. Mutant alleles could be reproducibly detected with a mutant-to-wild type ratio of 30% by direct sequencing and of 1% by PNA-clamping PCR.

### Detection of *KRAS* mutant subtypes using PNA-LNA mediated LAMP

**Fig 5:**
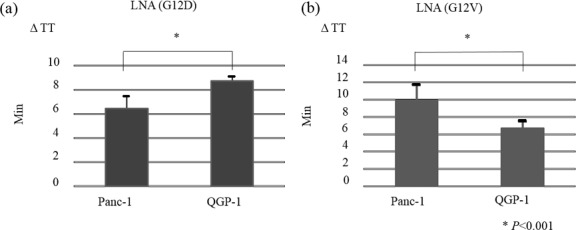
Detection of *KRAS* mutation subtypes using PNA-LNA mediated LAMP. (a) By using an LNA primer with a sequence complementary to that encoding the G12D mutation, cDNA from the Panc-1 cell line that expresses the *KRAS* G12D mutant was amplified more rapidly than cDNA from the QGP-1 cell line that expresses the *KRAS* G12V mutant (*P*<0.01). (b) Conversely, by using an LNA primer with a sequence complementary to that encoding the KRAS G12V mutation, cDNA from the QGP-1 cell line that expresses the *KRAS* G12V mutant was amplified more rapidly than cDNA from the Panc-1 cell line that expresses the *KRAS* G12D mutant (*P*<0.01). ΔTT is defined in the method.

When the LNA (G12D) primer was used for PNA-LNA mediated LAMP, ΔTT was significantly lower for the Panc-1 samples that express the G12D mutation than for the QGP-1 samples that express the G12V mutation. Conversely, when the LNA (G12V) primer was used for PNA-LNA mediated LAMP, ΔTT was significantly lower for the QGP-1 samples than for the Panc-1 samples (Fig 5).

## Discussions

The LAMP method is a non-PCR DNA amplification method that has the advantage of high sensitivity and a short reaction time. This is attributable to recognition of the target sequence by six independent sequences in the initial stage and by four independent sequences during the later stages of the LAMP reaction [18]. This method has been mostly applied to the detection of a small amount of DNA [22–26] and there have only been a few reports describing the use of the LAMP method for the detection of point mutations. Badolo et al. [27] reported rapid detection of the *kdr* mutation in mosquitos and Pan L et al. [29] used the LAMP assay to detect mutations in herbicide-resistant weeds. Both of these studies employed a 3’ mismatch on one LAMP primer, but this protocol in our hands failed to detect the *KRAS* point mutations. We concluded from our study that, even though there was mismatched hybridization between the LAMP primer and the cDNA, other LAMP primers that matched with the cDNA could amplify the cDNA. We subsequently developed a novel methodology for detection of *KRAS* mutation using two unique techniques, artificial DNAs (PNA and LNA) and the LAMP method. At the beginning of this study, we tried to apply a protocol similar to that of the PNA-clamping PCR method to LAMP, in which we designed conflicting PNA with wild type *KRAS* sequence between 2 LAMP primers or by one of the four LAMP primers, in order to stop the DNA amplification reaction of LAMP. However, by using this protocol, we could not get reproducible results among the samples. The reason we could not detect the point mutation is that even though the sequence of the PNA inhibited one of the four primers at the starting point of LAMP, the other three primers could amplify DNA, and thus PNA failed to suppress DNA amplification. In the next steps, we developed a different protocol in which we designed PNA with the addition of a self-annealing LNA primer. This self-annealing primer was in addition to the four primary primers in LAMP. A self-annealing primer has been previously used to decrease non-specific amplification in PCR [30]. In this new protocol, LAMP could not amplify DNA with the four primary primers, but could amplify DNA by addition of the fifth self-annealing primer. The competition between the PNA probe and the self-annealing primer could reproducibly detect the *KRAS* point mutation. Only one reported study, which was that of Minnucci et al., used PNA and LAMP to detect a point mutation [28]. Similar to our method, they used a self-annealing primer and successfully identified PML-RARA transcripts in acute promyelocytic leukemia. We have further advanced the protocol by adding LNA, and have developed the new PNA-LNA mediated LAMP method, which resulted in successful distinction of different *KRAS* mutant subtypes as well as detection of *KRAS* point mutation.

Compared with conventional methods, the PNA-LNA mediated LAMP method has multiple advantages. Firstly, the reaction time of PNA-LNA mediated LAMP, which is within 15 to 50 min, is shorter than that required by conventional methods to reach a final assessment of *KRAS* mutation status. Since the method is conducted at constant temperature, there is no time loss resulting from the temperature changes that are necessary for conventional PCR methods. Secondly, the LOD of PNA-LNA mediated LAMP was much lower than conventional methods. Mutant alleles could be reproducibly detected by PNA-LNA mediated LAMP in samples that were diluted down to a 0.1% mutant-to-wild type ratio. In contrast, Mutant alleles could be reproducibly detected with a mutant-to-wild type ratio of 30% by direct sequencing and of 1% by PNA-clamping PCR. Since clinical samples include a large amount of contaminating wild-type DNA, it is necessary to use highly sensitive methods for point mutation detection. In the past reports [8], the LOD of direct sequencing, PNA clamping PCR and Amplification refractory mutation system was 10–30%, 1–5% and 1–5%. PNA-LNA mediated LAMP appears to be a suitably high-sensitive method for detecting point mutations in clinical samples compared with other conventional methods. Thirdly, the amplified products of LAMP can be visually detected by adding a fluorescent detection reagent. Visualization of the LAMP products is convenient for a quick assay of mutation. Fourthly, PNA-LNA mediated LAMP does not need complicated equipment. Since it can be conducted under isothermal temperature conditions, only a standard laboratory water bath that can provide a constant temperature of 65°C, is required. Moreover, all the reagents used for this method are common and inexpensive. Therefore, this method is potentially more cost effective than conventional methods. Thus, this method may be useful even for minimally equipped laboratories and clinical institutes that are unfamiliar with molecular analysis methods. Finally, by using PNA-LNA mediated LAMP it is possible to detect different mutant subtypes. Generally, malignant tumors have some point mutation subtypes in the same gene. In colon cancer and pancreatic cancer, detection of *KRAS* mutant subtypes is extremely useful in predicting the therapeutic efficacy of chemotherapy and in identifying patients with poor prognosis [31].

## Conclusions

Given the advantages mentioned above, this novel methodology of PNA-LNA mediated LAMP significantly improved the overall efficiency of detection of point mutation. In the next step, we will apply this method to clinical samples. Clinical application of this method will allow assessment of the *KRAS* mutation status of each case immediately after sample biopsy, and thereby *KRAS* mutation status can be quickly connected to the appropriate treatment. Also, this method can be useful for population research such as molecular pathological epidemiology [32].
